# Supplementary material for: Detecting global and local hierarchical structures in cell-cell communication using CrossChat
Source: Nat Commun. 2024 Dec 3;15:10542. doi: 10.1038/s41467-024-54821-x (PMC11615294; doi:10.1038/s41467-024-54821-x)
Supplement: Supplementary file 1 — Supplementary Information [file 41467_2024_54821_MOESM1_ESM.pdf]

Supplementary Information

Detecting global and local hierarchical structures in cell-cell communication using CrossChat

Xinyi Wang[1], Axel A. Almet[1,2]*, Qing Nie[1,2,3]*

1 Department of Mathematics, University of California, Irvine, CA, United States

2 The NSF-Simons Center for Multiscale Cell Fate Research, University of California, Irvine, CA, United States

3 Department of Developmental and Cell Biology, University of California, Irvine, CA, United States

* Co-corresponding authors: aalmet@uci.edu, qnie@uci.edu

This file includes robustness analysis of CrossChatH, and Supplementary Figures 1-11.

Robustness of CrossChatH

We tested the robustness of hierarchical clustering of CrossChatH to selection of K in K-nearest neighbor graph construction, noise, sparsity, and normalization, on a PBMC dataset. To evaluate the robustness of hierarchical clustering with respect to K value in KNN graph construction, we ran the method with varying K values: 5, 10, 15, 20, 25, and 50. We calculate the multiscale similarity between clustering results generated by the different K values, and observed that the score between each multiscale clustering is almost always greater than 0.9, indicating a high robustness to K value selection. To evaluate the robustness of clusters to noise, we generated three scaled Gaussian noise and added them to the gene expression matrix. Specifically, the Gaussian noise is multiplied by 3 different scales: 10^{-3}, 10^{-4}, and 10^{-5}. The multiscale similarity between the clustering with added Gaussian noise and the clustering with no noise is still very high, indicating a high robustness of our clustering to noise. To evaluate the robustness to dropout, we chose four dropout rates: 0.1, 0.2, 0.3, and 0.5. The multiscale similarity decreases when dropout rate increases. Finally, to evaluate the robustness to different normalization methods, we tested three popular normalizations, including log-normalization, counts per million, and centered log-ratio transformation. We found that log-normalization-based and counts-per-million-based normalization produced similar multiscale clustering results, and while normalization using centered log-ratio transformation produced somewhat different results.

Evaluating multiscale similarity: For two multiscale clustering results, $X = \{X_1, X_2, \cdots, X_m\}$ and $Y = \{Y_1, Y_2, \cdots, Y_n\}$, where X_i denotes the communities detected by the first clustering results at scale i, and Y_j has an analogous definition. The multiscale-similarity between X and Y is defined as:

$$MSSim(X,Y) = \frac{1}{2}\left[\frac{1}{m}\sum_{i=1}^{m} min_{1\le j\le n} ARI(X_i, Y_j) + \frac{1}{n}\sum_{j=1}^{n} min_{1\le i\le m} ARI(X_i, Y_j)\right]$$

where $ARI(X_i, Y_j)$ is the Adjusted Rand Index between two clustering results X_i and Y_j.

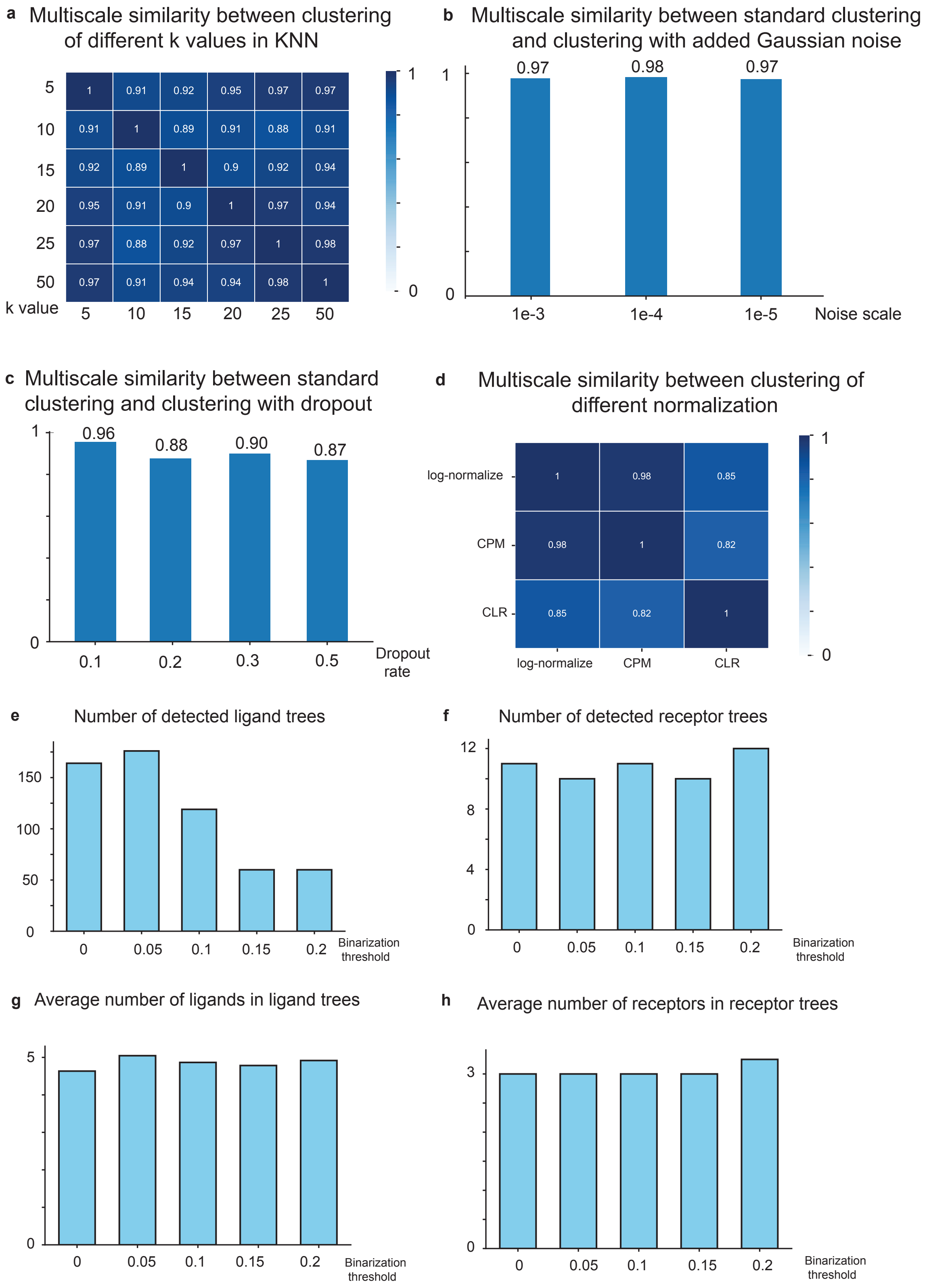

a
Multiscale similarity between clustering of different k values in KNN
5 10 15 20 25 50
1 0.91 0.92 0.95 0.97 0.97
0.91 1 0.89 0.91 0.88 0.91
0.92 0.89 1 0.9 0.92 0.94
0.95 0.91 0.9 1 0.97 0.94
0.97 0.88 0.92 0.97 1 0.98
0.97 0.91 0.94 0.94 0.98 1
k value
5 10 15 20 25 50
1
0
b
Multiscale similarity between standard clustering and clustering with added Gaussian noise
0.97
0.98
0.97
1
0
1e-3
1e-4
1e-5
Noise scale
c
Multiscale similarity between standard clustering and clustering with dropout
0.96
0.88
0.90
0.87
1
0
0.1
0.2
0.3
0.5
Dropout rate
d
Multiscale similarity between clustering of different normalization
log-normalize
CPM
CLR
1 0.98 0.85
0.98 1 0.82
0.85 0.82 1
log-normalize
CPM
CLR
1
0
e
Number of detected ligand trees
150
100
50
0
0 0.05 0.1 0.15 0.2
Binarization threshold
f
Number of detected receptor trees
12
8
4
0
0 0.05 0.1 0.15 0.2
Binarization threshold
g
Average number of ligands in ligand trees
5
0
0 0.05 0.1 0.15 0.2
Binarization threshold
h
Average number of receptors in receptor trees
3
0
0 0.05 0.1 0.15 0.2
Binarization threshold

Supplementary Fig. 1: Robustness test of CrossChatH with respect to different parameters. a Robustness test of CrossChatH with respect to selection of K in K-nearest neighbor algorithm. **b** Robustness test of CrossChatH with respect to noise. **c** Robustness test of CrossChatH with respect to dropout. **d** Robustness test of CrossChatH with respect to different normalization. **e** Number of detected ligand trees with varying threshold. **f** Number of detected receptor trees with varying threshold. **g** Average number of ligands in ligands trees under varying threshold. **h** Average number of ligands in receptors trees under varying threshold.Source data are provided as a Source Data file.

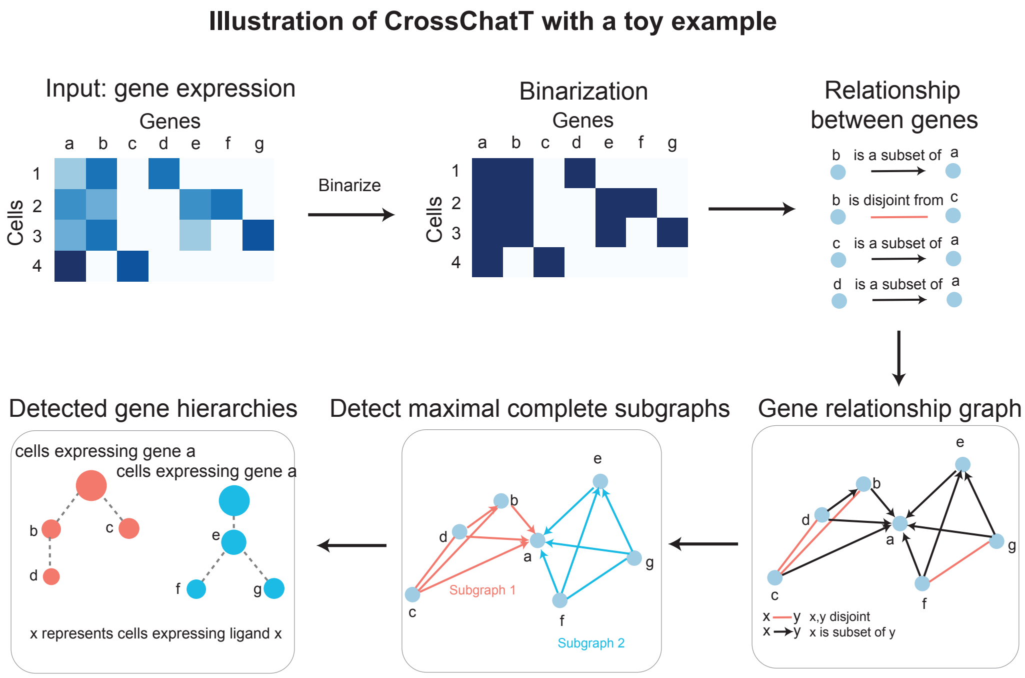

Supplementary Fig. 2: Detailed illustration of CrossChatT. First, binarization on the gene expression matrix is performed in order to find the support for each ligand/receptor. Next, extract relationship between genes based on the binarization matrix, and build gene relationship graph. Based on the gene relationship graph, all maximal complete subgraphs are detected. Each detected subgraph forms a detected gene hierarchy.

a Jaccard distance of multiscale clusters and cell types

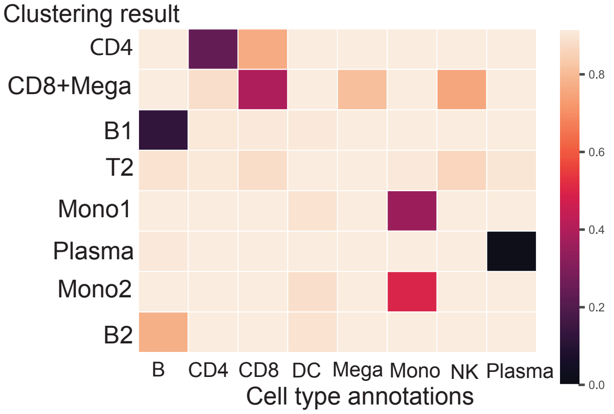

b Top 15 specific ligand-receptor pairs table

Order	Ligand	Receptor	Ligand cluster	Receptor cluster
1	CCL5	CCR1	CD8+Mega	Mono
2	ANXA1	FPR1	Mono	Mono
3	CCL5	CCR4	CD8+Mega	CD4
4	CCL5	CCR5	CD8+Mega	CD8+Mega
5	CCL5	ACKR2	CD8+Mega	T2
6	PSAP	GPR37L1	Mono	Mono1
7	APP	CD74	Mono1	B
8	GRN	SORT1	Mono	Mono1
9	LGALS9	IGHM	Mono	B
10	GZMA	F2R	CD8+Mega	CD8+Mega
11	GZMA	F2RL2	CD8+Mega	T2
12	GZMA	F2RL3	CD8+Mega	T2
13	GZMA	F2RL1	CD8+Mega	T2
14	CD99	CD99	CD8+Mega	CD8+Mega
15	CD99	PILRA	CD8+Mega	Mono

c Multiscale clustering using ligands

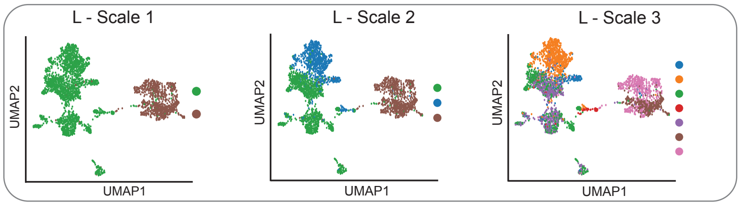

d Multiscale clustering using receptors

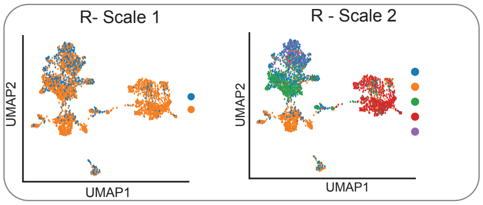

Supplementary Fig. 3: Application of CrossChatH to PBMC cells sampled from COVID-19 patients. a Jaccard distance between hierarchical clusters at finest scale and cell types. **b** Top 15 ligand-receptor pairs with highest specificity. Ligand cluster, receptor cluster correspond to their most specific ligand/receptor cluster. **c** Hierarchical clustering using only ligands as input gives three scales of clusters. **d** Hierarchical clustering using only receptors as input gives two scales of clusters.

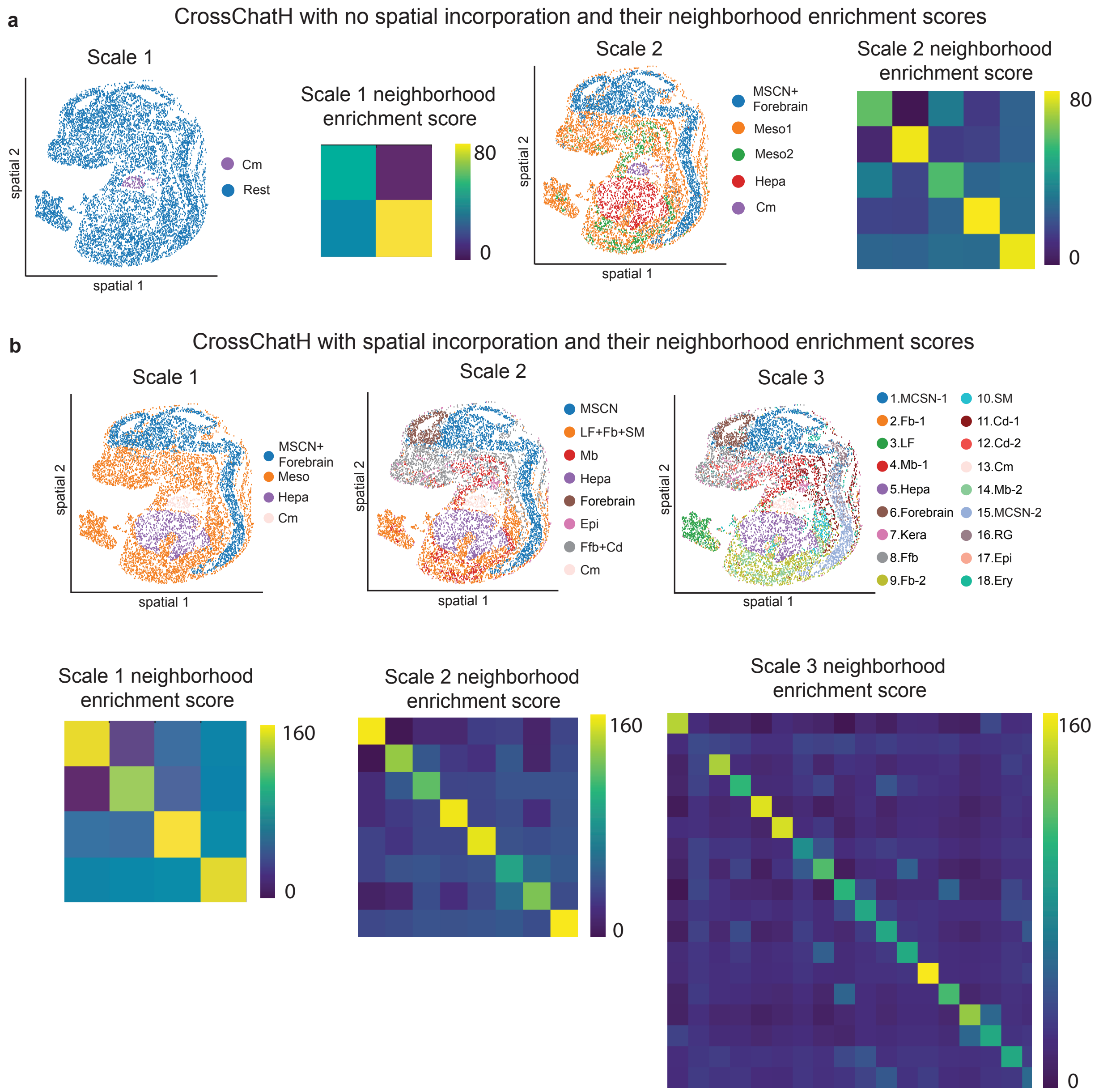

Supplementary Fig. 4 Demonstration of spatial incorporation for CrossChatH-S.
a CrossChatH with no spatial incorporation and their neighborhood enrichment scores **b** CrossChatH with spatial incorporation and their neighborhood enrichment scores. The detected clusters using CrossChatH-S which incorporates spatial information are more spatially coherent, compared to the clusters generated without spatial incorporation. This observation can be shown by higher neighborhood enrichment score of the spatial clusters detected by CrossChatH-S.

a

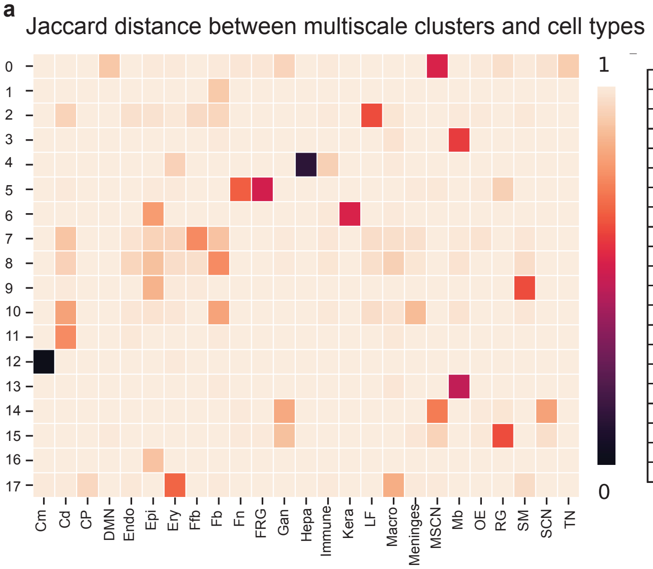

b

Top 20 specific ligand-receptor pairs

Order	Ligand	Receptor	Ligand cluster	Receptor cluster
1	*Igf2*	*Igf2r*	Meso	Meso
2	*Igf2*	*Igf1r*	Meso	Mb
3	*Nrxn1*	*Nlgn1*	MSCN	MSCN
4	*Nrxn1*	*Nlgn3*	MSCN	MSCN+Forebrain
5	*Nrxn1*	*Nlgn2*	MSCN	MSCN
6	*Dlk1*	*Notch2*	Meso	Meso
7	*Dlk1*	*Notch1*	Meso	Kera
8	*Dlk1*	*Notch3*	Meso	Meso
9	*Ncam1*	*Ncam1*	MSCN	MSCN
10	*Mdk*	*Ncl*	Meso	Meso
11	*Mdk*	*Ptprz1*	Meso	Meso
12	*Nrxn3*	*Nlgn1*	MSCN+Forebrain	MSCN
13	*Spp1*	*Cd44*	Cd-2	Meso
14	*Mdk*	*Lrp1*	Meso	Meso
15	*Nrg3*	*Erbb4*	MSCN	MSCN
16	*Mdk*	*Sdc1*	Meso	Epi
17	*Mdk*	*Sdc2*	Meso	Meso
18	*Mdk*	*Alk*	Meso	MSCN
19	*Mdk*	*Sdc4*	Meso	Ffb+Cd
20	*Lgals9*	*Cd44*	Epi	Meso

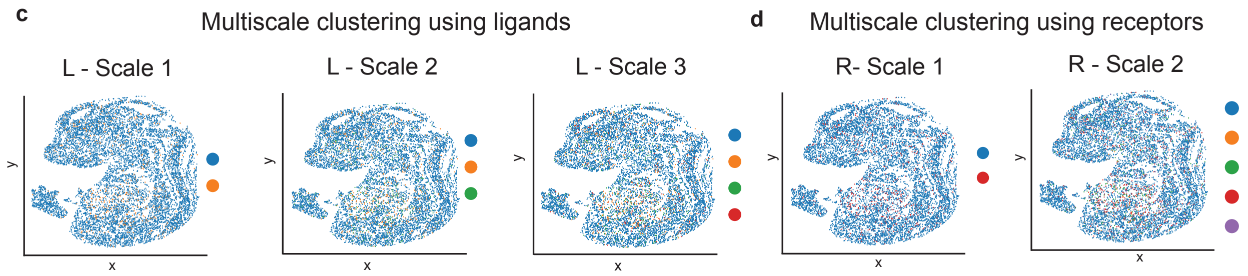

Supplementary Fig. 5: Application of CrossChatH-S to Stereo-seq mouse embryo at day E16.5 data. **a** Jaccard distance between hierarchical clusters at finest scale and cell types of mouse embryo at day E16.5. **b** Top 20 ligand-receptor pairs with highest specificity. Ligand cluster, receptor cluster correspond to their most specific ligand/receptor cluster. **c** Hierarchical clustering using only ligands as input gives three scales of clusters. **d** Hierarchical clustering using only receptors as input gives two scales of clusters.

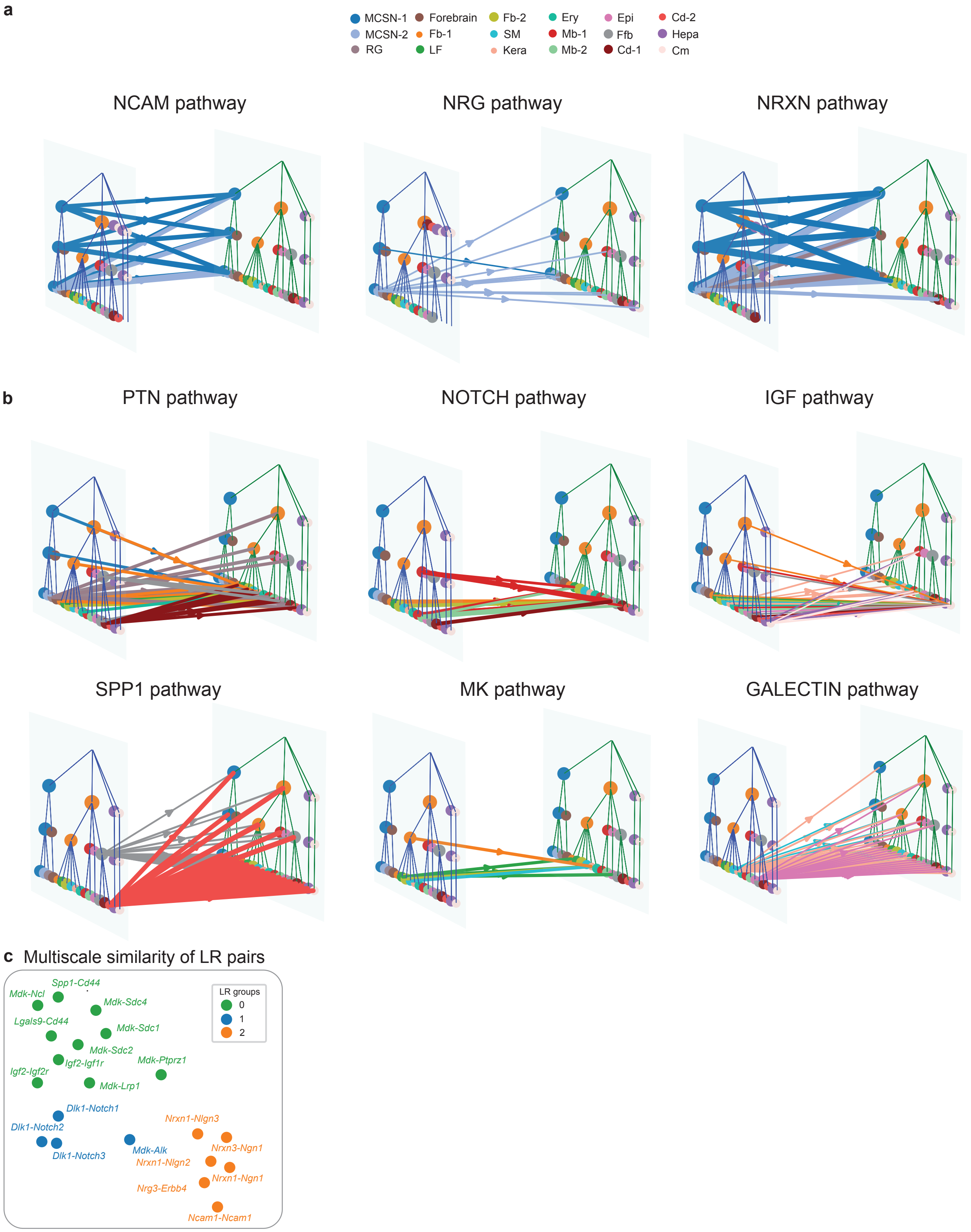
a
MCSN-1
MCSN-2
RG
Forebrain
Fb-1
LF
Fb-2
SM
Kera
Ery
Mb-1
Mb-2
Epi
Ffb
Cd-1
Cd-2
Hepa
Cm
NCAM pathway
NRG pathway
NRXN pathway
b
PTN pathway
NOTCH pathway
IGF pathway
SPP1 pathway
MK pathway
GALECTIN pathway
c Multiscale similarity of LR pairs
LR groups
0
1
2
Mdk-Ncl
Spp1-Cd44
Mdk-Sdc4
Lgals9-Cd44
Mdk-Sdc1
Mdk-Sdc2
Igf2-Igf1r
Mdk-Ptprz1
Igf2-Igf2r
Mdk-Lrp1
Dlk1-Notch1
Dlk1-Notch2
Dlk1-Notch3
Mdk-Alk
Nrxn1-Nlgn3
Nrxn3-Ngn1
Nrxn1-Nlgn2
Nrxn1-Ngn1
Nrg3-Erbb4
Ncam1-Ncam1

Supplementary Fig. 6: Cell-cell communications of pathways on Stereo-seq mouse embryo at day E16.5 and multiscale similarity of ligand-receptor interactions. a Cell-cell communications under Neural Cell Adhesion Molecule (NCAM), Neuregulin (NRG), Neurexin (NRXN) pathways. These pathways are associated with signaling mainly sent from brain and spinal cord neurons. **b** Cell-cell communications under Pleiotrophin (PTN), NOTCH, Insulin-like growth factor (IGF), Secreted phosphoprotein 1 (SPP1), Midkine (MK), GALECTIN pathways. These pathways are associated with signaling mainly sent from the rest of cells other than neurons. **c** Similarity of top 20 most specific ligand-receptor interactions under hierarchical clusters. Group 1 include interactions from MDK and IGF pathways, performing cell growth functions. Group 2 mainly contains interactions from *Dlk1* to *Notch* receptors. Group 3 contains signaling within neurons.

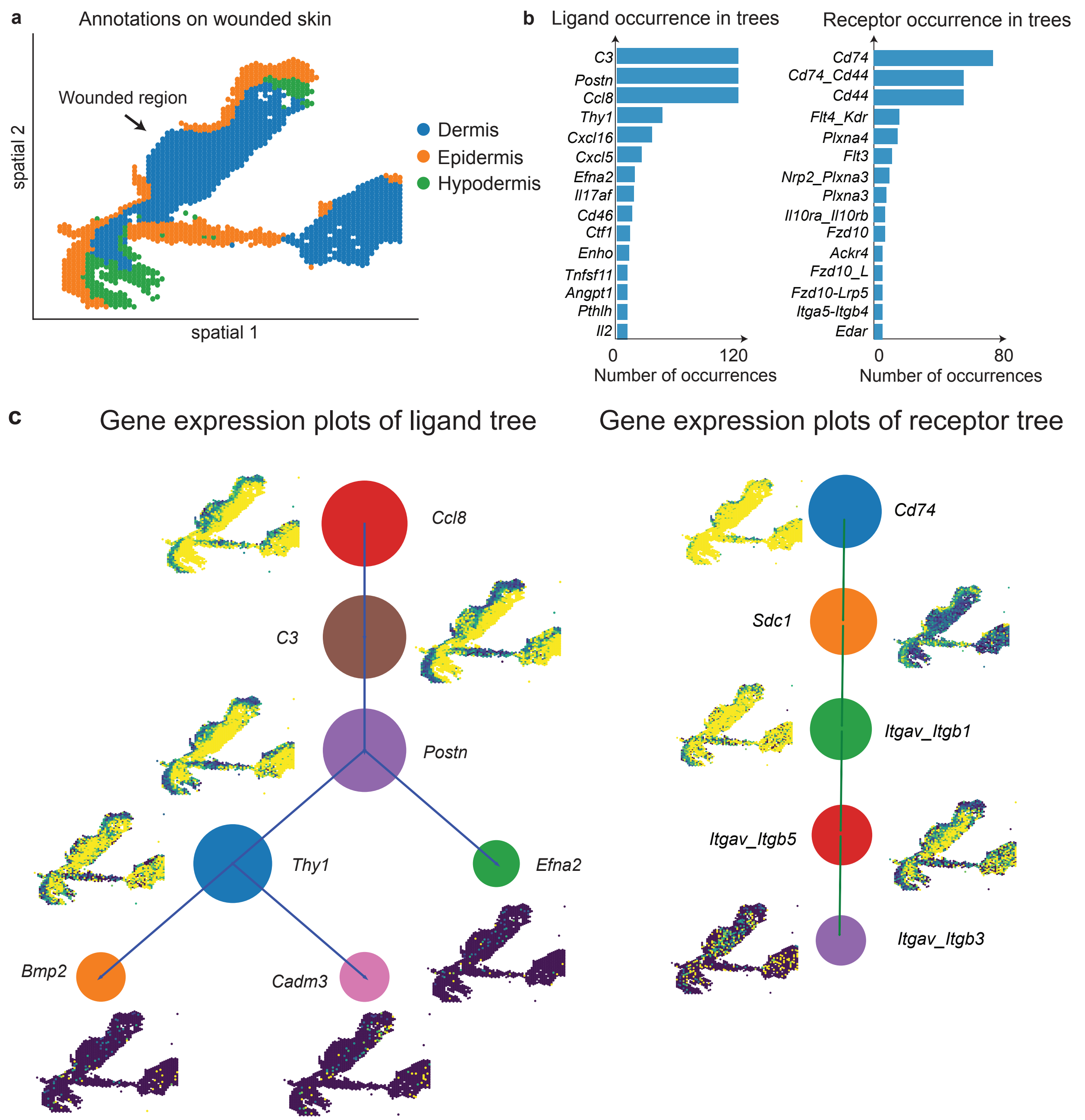

Supplementary Fig. 7: Cell-cell communication inferred from 10x Visium mouse skin multiscale similarity of ligand-receptor interactions. **a** Annotations of cells of Visium mouse skin data at post-wound day 7. **b** Ligands/receptors occurrence in ligands/receptors trees detected using CrossChatT-S. **c** Gene expression of ligands and receptors in interaction tree 1 (see Fig. 6f). Cells expressing ligands/receptors at the top

include cells containing ligands/receptors at the bottom. Cells expressing ligands/receptors at the same scale are disjoint of each other.

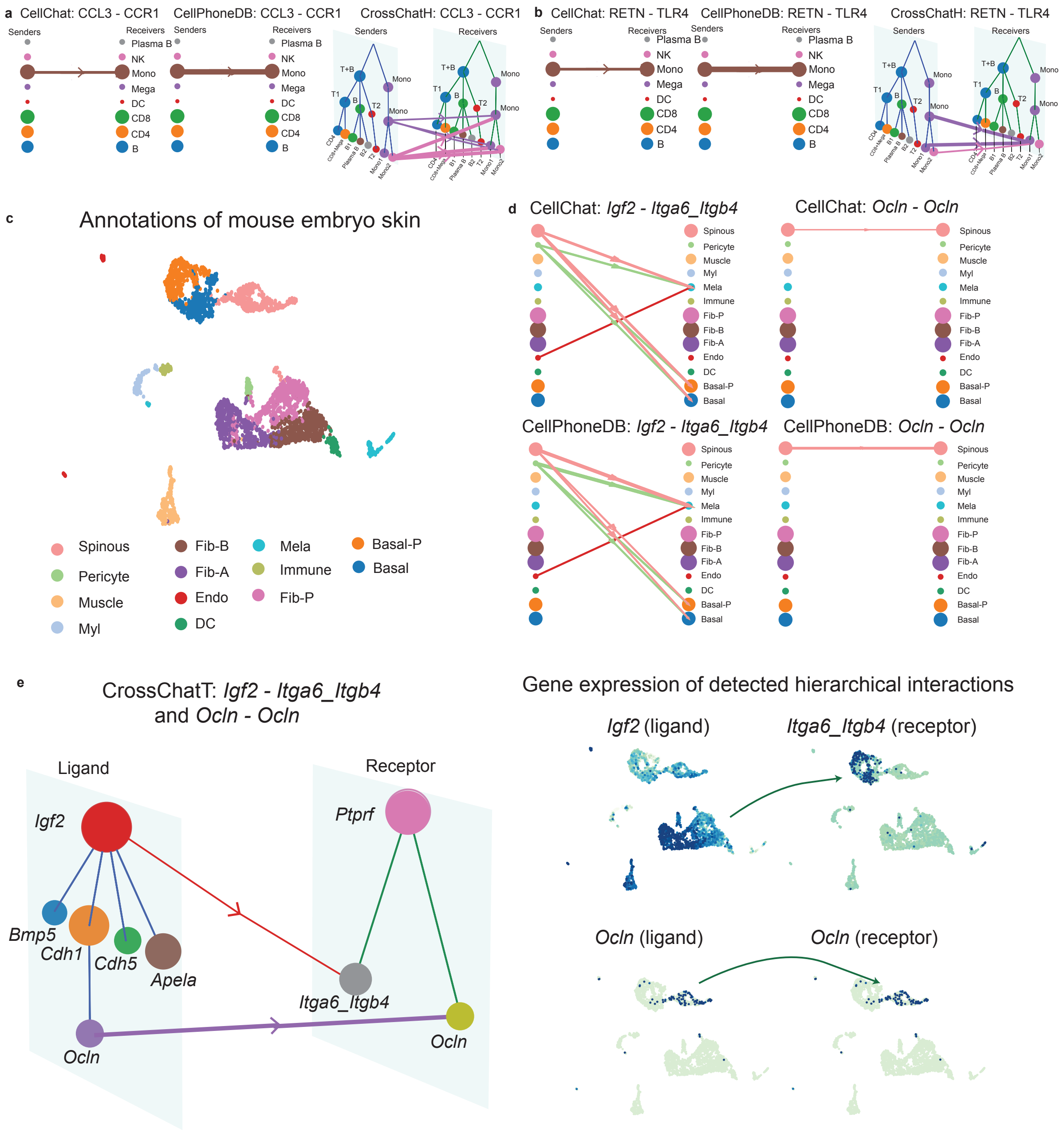

Supplementary Fig. 8: Comparison of CrossChat with existing CCC methods. **a** CrossChat detects more precise signaling by assigning CCL3-CCR1 signaling to a subset of monocytes, Mono2. **b** CrossChat detects more precise signaling by assigning the receivers of RETN-TLR4 signaling to a subset of monocytes, Mono1. **c** Cell type annotations of a mouse embryo skin data. **d** Visualization of *Igf2-Itga6_Itgb4, Ocln-Ocln* interactions calculated using CellChat. **e** Visualization of *Igf2-Itga6_Itgb4, Ocln-Ocln* interactions calculated using CrossChatT.

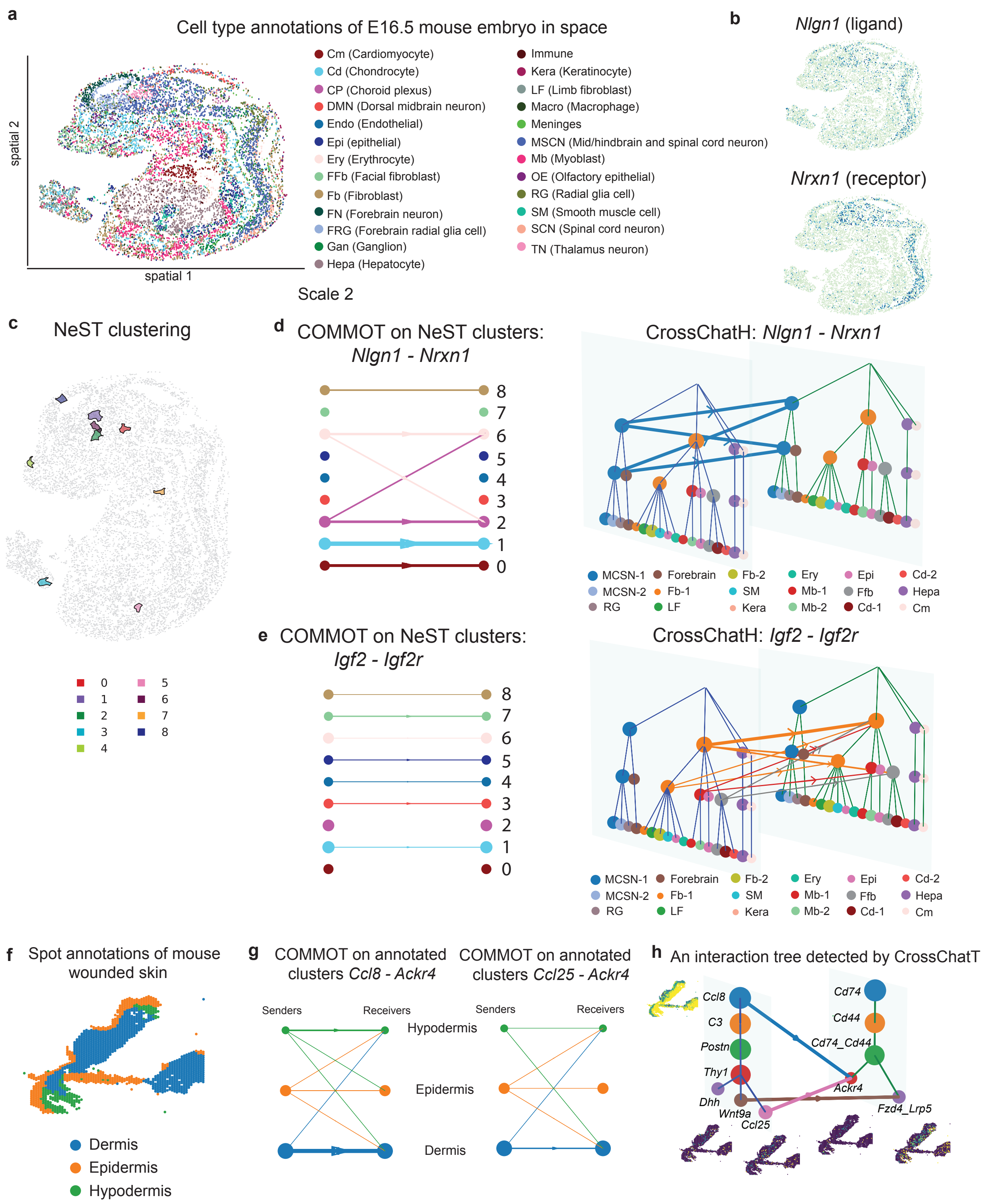

Supplementary Fig. 9: Comparison of CrossChat-S with existing spatial CCC methods. a Cell type annotations of spatial mouse embryo dataset in space. **b** Gene expression of *Nlgn1* ligand and *Nrxn1* receptor in space. **c** Spatial clusters detected by

NeST. **d** left: Visualization of *Nlgn1-Nrxn1* interactions on NeST clusters calculated using COMMOT. right: Visualization of *Nlgn1-Nrxn1* interactions on CrossChatH clusters calculated using COMMOT. **e** left: Visualization of *Igf2-Igf2r* interactions on NeST clusters calculated using COMMOT. right: Visualization of *Igf-Igf2r* interactions on CrossChatH clusters calculated using COMMOT. **f** Spot annotations of mouse wounded skin data in space. **g** Interaction of *Ccl8–Ackr4, Ccl25–Ackr4* calculated using COMMOT. **h** An interaction tree detected by CrossChatT that includes *Ccl8–Ackr4 and Ccl25–Ackr4* signaling.

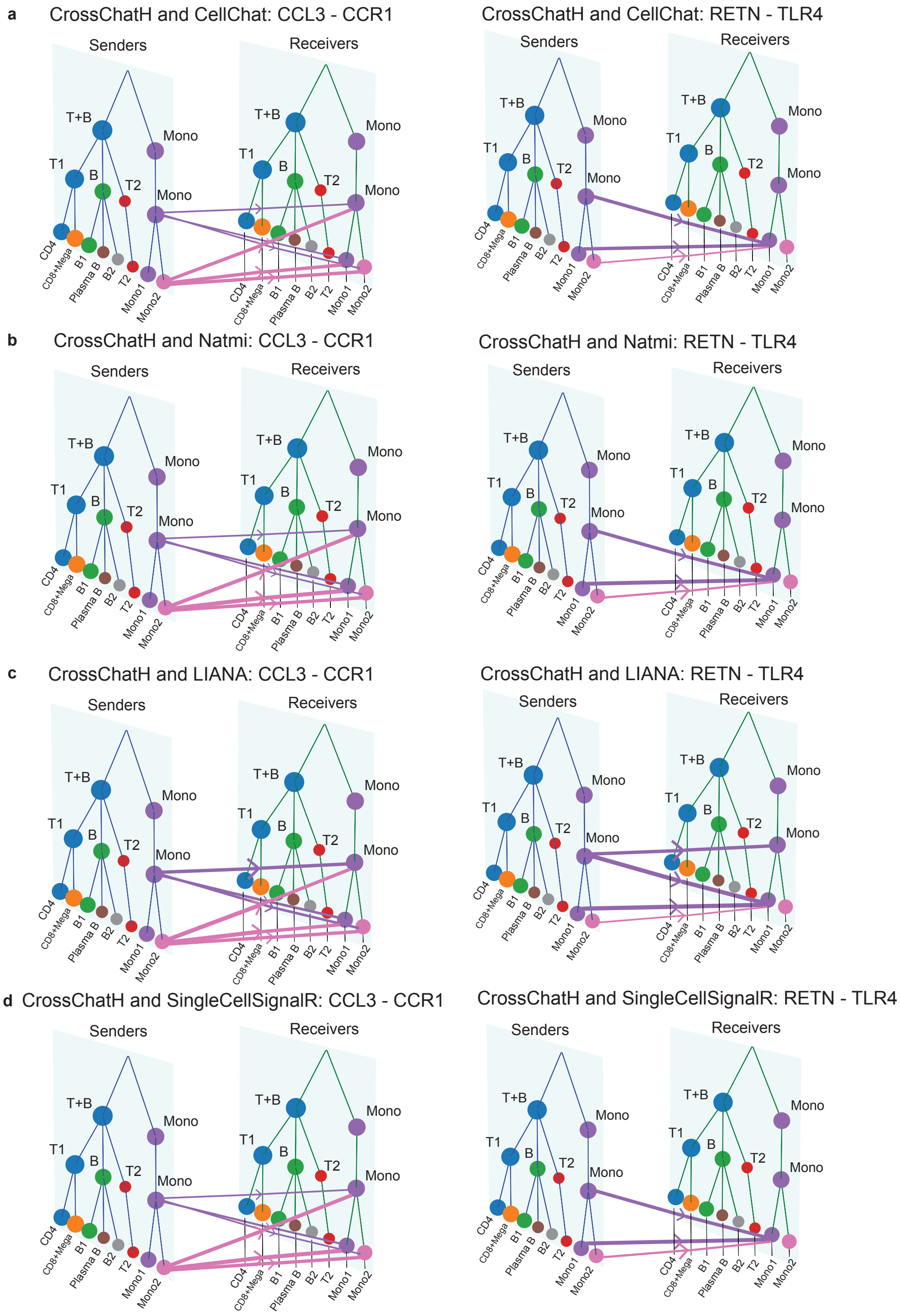

Supplementary Fig. 10: Comparison of CrossChatH analysis with different CCC packages. a CCC detected by CrossChatH using CellChat **b** CCC detected by CrossChatH using NATMI **c** CCC detected by CrossChatH using LIANA **d** CCC detected by CrossChatH using SingleCellSignalR

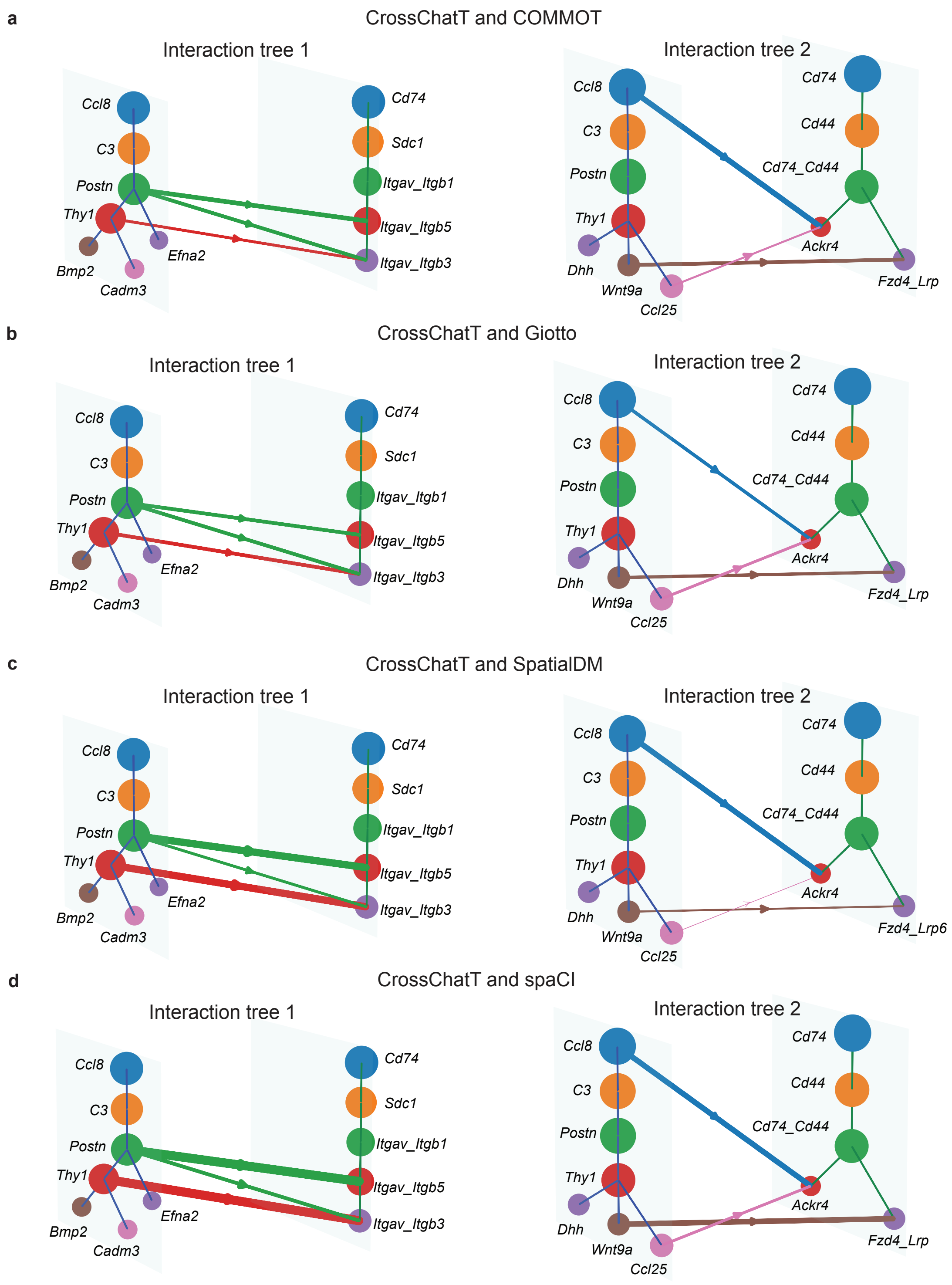

Supplementary Fig. 11 Comparison of CrossChatT analysis with different CCC methods. **a** CCC detected by CrossChatH using COMMOT **b** CCC detected by CrossChatH using Giotto **c** CCC detected by CrossChatH using SpatialDM **d** CCC detected by CrossChatH using spaCI.

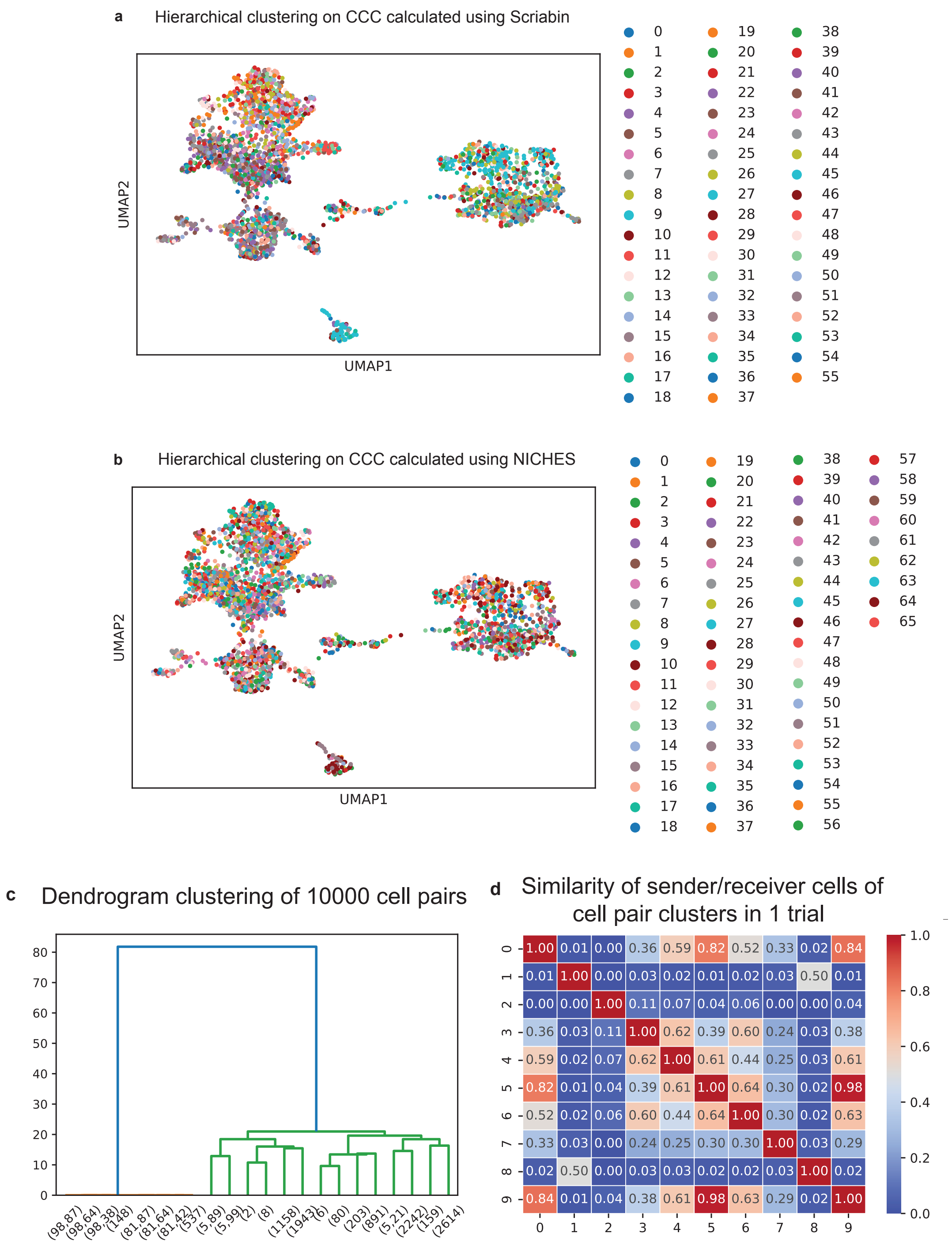

Supplementary Fig. 12: Hierarchical clustering on CCC calculated using single-cell level inference tools. a CrossChatH clustering on CCC calculated using Scriabin **b** CrossChatH clustering on CCC calculated using NICHES **c** Dendrogram clustering of 10000 cell pairs of COVID-19 PBMC cells **d** Jaccard similarity of sender/receiver cells of cell pair clusters. Source data are provided as a Source Data file.
